# Impact of Obstructive Sleep Apnea and Triglyceride Glucose Index on Cardiovascular Events in Acute Coronary Syndrome Patients: A Post-Hoc Analysis of the OSA–ACS Study

**DOI:** 10.31083/RCM36205

**Published:** 2025-05-21

**Authors:** Yuekun Zhang, Ding Xu, Wen Zheng, Wen Hao, Lei Zhen, Yan Yan, Xiao Wang, Shaoping Nie

**Affiliations:** ^1^Center for Coronary Artery Disease, Division of Cardiology, Beijing Anzhen Hospital, Capital Medical University, 100029 Beijing, China; ^2^Cardiometabolic Medicine Center, Fuwai Hospital, National Center for Cardiovascular Diseases, Chinese Academy of Medical Sciences and Peking Union Medical College, 100037 Beijing, China

**Keywords:** acute coronary syndrome, insulin resistance, obstructive sleep apnea, triglyceride glucose index

## Abstract

**Background:**

Obstructive sleep apnea (OSA) is highly prevalent in patients with acute coronary syndrome (ACS). The triglyceride glucose (TyG) index is considered closely linked to cardiovascular risk. However, the relationship between OSA, TyG index, and cardiovascular outcomes in ACS patients remains unclear. Hence, this study aimed to examine the effects of OSA and the TyG index on cardiovascular outcomes in ACS patients.

**Methods:**

This post-hoc analysis included 1853 patients from the OSA–ACS project, a single-center prospective cohort study that enrolled ACS patients admitted between January 2015 and December 2019. OSA was defined as an apnea–hypopnea index of ≥15 events/hour. The primary endpoint was major adverse cardiovascular and cerebrovascular events (MACCE). Multivariable Cox regression models were used to evaluate the impact of OSA on cardiovascular events across the TyG index categories.

**Results:**

OSA was present in 52.5% of the participants, with a mean TyG index of 9.02 ± 0.68. Over a median follow-up of 35.1 (19.0–43.5) months, OSA was significantly associated with a heightened risk of MACCE (adjusted hazard ratio (aHR): 1.556; 95% confidence interval (CI): 1.040–2.326; *p* = 0.031) in the high TyG group within the fully adjusted model, along with elevated risk of hospitalization for unstable angina (aHR: 1.785; 95% CI: 1.072–2.971;* p* = 0.026). No significant associations were observed between OSA and MACCE in the low and moderate TyG groups.

**Conclusions:**

This analysis demonstrates that OSA significantly increases the risk of adverse cardiovascular events in ACS patients with a high TyG index, underscoring the importance of routine OSA screening in these high-risk ACS patients to optimize cardiovascular risk stratification and personalize treatment strategies.

**The Clinical Trial Registration:**

NCT03362385, https://clinicaltrials.gov/expert-search?term=NCT03362385.

## 1. Introduction 

Obstructive sleep apnea (OSA) is a prevalent sleep disorder, characterized by intermittent complete or partial upper airway obstruction, affecting 40% to 80% of individuals with cardiovascular diseases. This condition leads to intermittent hypoxemia, sleep fragmentation, significant negative intrathoracic pressure swings, and alterations in the gut microbiota, ultimately increasing the risk of cardiovascular events, such as unstable angina (UA), sudden cardiac death, and acute myocardial infarction [1, 2, 3, 4]. Previous studies have demonstrated that OSA exacerbates insulin resistance, thereby contributing to the progression of cardiovascular diseases [5, 6, 7].

The triglyceride glucose (TyG) index, a biomarker calculated from fasting triglyceride and glucose levels, has been acknowledged as a reliable and non-invasive indicator of insulin resistance [8]. This index effectively integrates lipid and glucose metabolism, enhancing cardiovascular risk prediction [9, 10, 11]. Previous meta-analyses have demonstrated associations between elevated TyG index and increased risks of heart failure (HF) [12], peripheral arterial disease [13], and hypertension [14]. Furthermore, studies have demonstrated that the TyG index is significantly higher in patients with OSA compared to non-OSA individuals, with elevated TyG index independently linked to both increased risk and severity of OSA, even after adjusting for potential confounding factors [15, 16, 17]. For example, in non-obese and non-diabetic patients, Bikov *et al*. [18] revealed that the TyG index was associated with OSA and its severity.

Given the heterogeneity of OSA, which contributes to the variable efficacy of continuous positive airway pressure (CPAP) interventions and its impact on prognosis, identifying high-risk populations likely to benefit from targeted intervention for OSA is essential [19, 20]. To date, research has yet to investigate the influence of OSA on the prognosis of patients with acute coronary syndrome (ACS) stratified by the TyG index. Therefore, we conducted a post-hoc analysis to evaluate the effects of OSA and TyG index on cardiovascular risk among patients with ACS.

## 2. Materials and Methods

### 2.1 Study Population

The OSA-ACS project (NCT03362385) is a single-center prospective cohort study designed to evaluate the impact of obstructive sleep apnea on cardiovascular events in patients with ACS. This study enrolled ACS patients aged 18 to 85 who were hospitalized at the Beijing Anzhen Hospital between January 2015 and December 2019. Participants were excluded if they experienced cardiogenic shock, cardiac arrest, malignancies, failed sleep studies or recordings of less than 180 minutes, central sleep apnea, loss to follow-up, or regular CPAP therapy [21, 22]. The TyG index was derived using the following formula: TyG index = ln[fasting triglycerides (mg/dL) × fasting glucose (mg/dL)/2] [23, 24]. Written informed consent was obtained from all participants. This study adhered to the Declaration of Helsinki and received approval from the local committee (approval number: 2013025). The post-hoc analysis followed the Strengthening the Reporting of Observational Studies in Epidemiology (STROBE) guidelines.

### 2.2 Procedure and Management

Following clinical stabilization, nocturnal sleep assessments were conducted using a portable cardiopulmonary polygraphy device (ApneaLink, ResMed, Sydney, New South Wales, Australia), with a minimum recording requirement of 3 hours. Monitoring parameters included thoracoabdominal movement, nasal airflow, arterial oxygen saturation, and snoring. Sleep study was performed following the standards of the American Academy of Sleep Medicine [25]. Apnea was defined as an airflow cessation for ≥10 seconds. Hypopnea was defined as a ≥30% reduction in airflow lasting ≥10 seconds, accompanied by a ≥4% decrease in oxygen saturation. The apnea-hypopnea index (AHI) was calculated as the total number of apnea and hypopnea events per hour of recorded time. All sleep study data were independently reviewed by two sleep technologists, with discrepancies resolved by a senior sleep medicine consultant. Patients with an AHI ≥15 events/hour were assigned to the OSA group, while those with an AHI <15 events/hour were categorized as non-OSA.

All patients received guideline-recommended treatment. Unless contraindicated, dual antiplatelet therapy was prescribed for at least one year post-discharge. Patients diagnosed with moderate-to-severe OSA (AHI ≥15 events per hour), especially those with pronounced daytime sleepiness, were referred for comprehensive evaluation and potential intervention.

### 2.3 Follow-Up and Outcomes

Patients were followed up at 1 month, 3 months, 6 months, 1 year, and thereafter at every 6-month intervals through outpatient visits or telephone interviews. The primary endpoint was major adverse cardiovascular and cerebrovascular events (MACCE), comprising hospitalization for UA or HF, stroke, myocardial infarction, cardiovascular death, and ischemia-driven revascularization. Secondary endpoints included each individual component of MACCE, all repeat revascularizations, a composite of cardiovascular death, myocardial infarction, and ischemic stroke, as well as a composite of cardiac events excluding stroke.

### 2.4 Statistical Analysis

Variables following a normal distribution are presented as mean ± standard deviation and were compared using one-way analysis of variance (ANOVA). Data not following a normal distribution are reported as median (interquartile range) and analyzed via the Kruskal-Wallis test. Categorical data are expressed as frequencies and percentages, and differences are evaluated using the chi-square test. After stratifying by the level of TyG index, a Cox proportional hazards model was employed to evaluate the impact of OSA on cardiovascular events. Covariates were selected based on data characteristics and prior literature, utilizing three models for analysis: (1) an unadjusted model, (2) a model partially adjusted for the confounding covariates of age and sex, and (3) a fully adjusted model that includes age, sex, body mass index (BMI), estimated glomerular filtration rate (eGFR), left ventricular ejection fraction, diabetes, hypertension, prior stroke, prior myocardial infarction, smoking, diagnosis, presence of HF, coronary artery bypass grafting (CABG), P2Y12 inhibitors, and β-blockers. Statistical analyses were conducted using SPSS software (version 27.0, IBM SPSS Inc., Armonk, NY, USA), with a two-sided *p*-value of <0.05 considered statistically significant.

## 3. Results

**Fig. 1.**
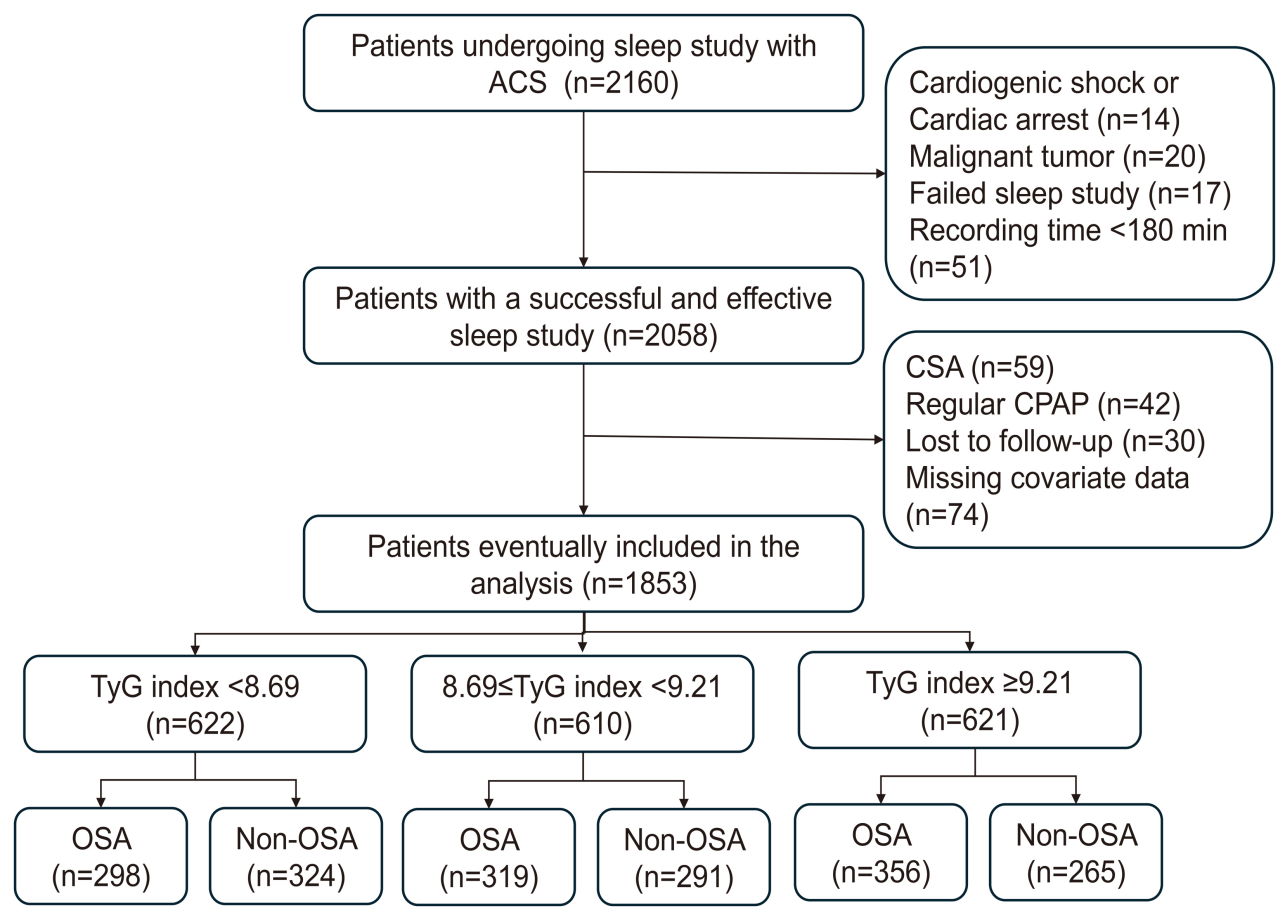
**Study flowchart**. ACS, acute coronary syndrome; CPAP, continuous positive airway pressure; CSA, central sleep apnea; OSA, obstructive sleep apnea; TyG, triglyceride glucose.

This analysis included 1853 ACS patients, of whom 52.5% (973/1853) had OSA, with a mean TyG index of 9.02 ± 0.68 (Fig. 1). Patients were categorized into three subgroups based on TyG index tertiles: high TyG group (TyG ≥ 9.21), moderate TyG group (8.69 ≤ TyG < 9.21), and low TyG group (TyG <8.69).

Participants in this study had a mean age of 56.4 ± 10.5 years, with male patients comprising 84.8% (1571/1853) of the cohort. As the TyG index increased, significant elevations were observed in BMI, neck circumference, and high-sensitivity C-reactive protein (hs-CRP) levels (*p* < 0.001). Additionally, patients in the high TyG group had significantly higher proportions of diabetes, hypertension, and hyperlipidemia (*p* < 0.05) and were younger compared with the moderate and low TyG groups (53.8 ± 10.5 vs. 57.1 ± 9.9 vs. 58.2 ± 10.5, *p* < 0.001). Further details on clinical characteristics are provided in Table 1 and **Supplementary Table 1**.

**Table 1.** **Baseline clinical characteristics by TyG index categories**.

		All	TyG index <8.69	8.69 ≤ TyG index < 9.21	TyG index ≥9.21	*p *value
		(N = 1853)	(N = 622)	(N = 610)	(N = 621)	
Demographics						
	Age, years	56.4 ± 10.5	58.2 ± 10.5	57.1 ± 9.9	53.8 ± 10.5	<0.001
	Male	1571 (84.8)	533 (85.7)	503 (82.5)	535 (86.2)	0.146
	BMI, kg/m^2^	26.8 ± 3.6	26.2 ± 3.6	27.0 ± 3.6	27.9 ± 3.6	<0.001
	Waist-to-hip ratio	0.98 (0.95–1.02)	0.97 (0.94–1.00)	0.98 (0.95–1.01)	0.99 (0.96–1.03)	<0.001
	Neck circumference, cm	41 (38–43)	40 (37–42)	41 (38–43)	42 (39–44)	<0.001
	Systolic BP, mmHg	126 (117–138)	127 (118–139)	126 (117–137)	127 (118–139)	0.513
	Diastolic BP, mmHg	76 (70–85)	76 (70–83)	75 (70–82)	78 (70–87)	<0.001
Medical History						
	Diabetes	590 (31.8)	120 (19.3)	170 (27.9)	300 (48.3)	<0.001
	Hypertension	1198 (64.7)	380 (61.1)	387 (63.4)	431 (69.4)	0.007
	Hyperlipidemia	607 (32.8)	190 (30.5)	181 (29.7)	236 (38.0)	0.003
	Family history of premature CAD	101 (5.5)	35 (5.6)	30 (4.9)	36 (5.8)	0.772
	Prior stroke	198 (10.7)	60 (9.6)	81 (13.3)	57 (9.2)	0.039
	Prior myocardial infarction	302 (16.3)	100 (16.1)	105 (17.2)	97 (15.6)	0.739
	Prior PCI	383 (20.7)	131 (21.1)	118 (19.3)	134 (21.6)	0.599
Smoking						0.057
	No	632 (34.1)	212 (34.1)	224 (36.7)	196 (31.6)	
	Current	878 (47.4)	285 (45.8)	270 (44.3)	323 (52.0)	
	Previous	343 (18.5)	125 (20.1)	116 (19.0)	102 (16.4)	
Drinking						0.004
	No	1137 (61.4)	385 (61.9)	386 (63.3)	366 (58.9)	
	Current	615 (33.2)	209 (33.6)	177 (29.0)	229 (36.9)	
	Previous	101 (5.5)	28 (4.5)	47 (7.7)	26 (4.2)	
Presence of HF		11 (0.6)	2 (0.3)	3 (0.5)	6 (1.0)	0.309
Baseline Tests						
	eGFR, mL/min/1.73 m^2^	105.2 (89.5–121.4)	106.5 (90.6–124.0)	104.9 (89.7–119.9)	104.1 (88.5–119.9)	0.151
	hs-CRP, mg/L	2.0 (0.8–6.1)	1.4 (0.5–5.3)	1.9 (0.8–5.8)	2.9 (1.1–6.9)	<0.001
	LVEF, %	61 (56–65)	62 (56–65)	62 (56–65)	61 (56–65)	0.502
	TyG index	9.02 ± 0.68	8.35 ± 0.29	8.94 ± 0.15	9.76 ± 0.51	<0.001

BMI, body mass index; BP, blood pressure; CAD, coronary artery disease; eGFR, estimated glomerular filtration rate; HF, heart failure; hs-CRP, high-sensitivity C-reactive protein; LVEF, left ventricular ejection fraction; PCI, percutaneous coronary intervention; TyG, triglyceride glucose.

In patients with a high TyG index, the prevalence of OSA was substantially elevated compared with the moderate and low TyG groups (57.3% vs. 52.3% vs. 47.9%, *p* = 0.004). These patients exhibited notably elevated median AHI, oxygen desaturation index (ODI), and prolonged duration of <90% oxygen saturation (*p* < 0.001), suggesting that OSA severity increases in parallel with TyG index. Patients in the high TyG group demonstrated a higher frequency of percutaneous coronary intervention (*p* = 0.009) and use of β-blockers (*p* = 0.004). Details on clinical presentations and management characteristics are provided in Table 2 and **Supplementary Table 2**.

**Table 2.** **Clinical presentations and management by TyG index categories**.

		All	TyG index <8.69	8.69 ≤ TyG index <9.21	TyG index ≥9.21	*p *value
		(N = 1853)	(N = 622)	(N = 610)	(N = 621)	
Diagnosis						0.283
	STEMI	418 (22.6)	132 (21.2)	150 (24.6)	136 (21.9)	
	NSTEMI	351 (18.9)	108 (17.4)	114 (18.7)	129 (20.8)	
	UA	1084 (58.5)	382 (61.4)	346 (56.7)	356 (57.3)	
Procedures						
	Coronary angiography	1806 (97.5)	606 (97.4)	591 (96.9)	609 (98.1)	0.418
	PCI	1164 (62.8)	363 (58.4)	387 (63.4)	414 (66.7)	0.009
	DES use	1007 (86.5)	308 (84.8)	343 (88.6)	356 (86.0)	0.294
	Baseline TIMI 0 or 1	404 (34.7)	115 (31.7)	133 (34.4)	156 (37.7)	0.212
	CABG	127 (6.9)	41 (6.6)	40 (6.6)	46 (7.4)	0.799
Sleep Study						
	OSA	973 (52.5)	298 (47.9)	319 (52.3)	356 (57.3)	0.004
	AHI, events·h^−⁢1^	15.8 (8.0–29.9)	14.1 (7.5–27.5)	15.7 (7.9–28.4)	18.5 (8.9–36.2)	<0.001
	ODI, events·h^−⁢1^	16.2 (8.8–28.5)	14.3 (8.2–25.5)	16.2 (8.6–27.7)	18.2 (9.6–32.5)	<0.001
	Nadir SaO_2_, %	85 (81–88)	86 (82–89)	85 (81–88)	85 (80–88)	0.002
	Mean SaO_2_, %	94 (93–95)	94 (93–95)	94 (93–95)	94 (93–95)	0.037
	Time with SaO_2_ <90%, %	2.1 (0.4–10.0)	1.8 (0.3–8.0)	3.0 (0.3–10.0)	3.0 (0.5–12.0)	0.001
	Epworth sleepiness scale	7.0 (4.0–11.0)	6.0 (3.0–10.0)	7.0 (4.0–11.0)	8.0 (5.0–12.0)	<0.001
Medications on Discharge						
	Aspirin	1805 (97.4)	600 (96.5)	597 (97.9)	608 (97.9)	0.190
	P2Y_12_ inhibitors	1702 (91.9)	557 (89.5)	568 (93.1)	577 (92.9)	0.036
	β-Blockers	1429 (77.1)	452 (72.7)	478 (78.4)	499 (80.4)	0.004
	ACEIs/ARBs	1151 (62.1)	365 (58.7)	395 (64.8)	391 (63.0)	0.078
	Statins	1825 (98.5)	615 (98.9)	602 (98.7)	608 (97.9)	0.333

ACEI, angiotensin-converting enzymes inhibitor; AHI, apnea-hypopnea index; ARB, angiotensin receptor blocker; CABG, coronary artery bypass grafting; DES, drug eluting stent; NSTEMI, non-ST-segment elevation myocardial infarction; ODI, oxygen desaturation index; OSA, obstructive sleep apnea; PCI, percutaneous coronary intervention; SaO_2_, arterial oxygen saturation; STEMI, ST-segment-elevation myocardial infarction; TIMI, thrombolysis in myocardial infarction; TyG, triglyceride glucose; UA, unstable angina.

**Fig. 2.**
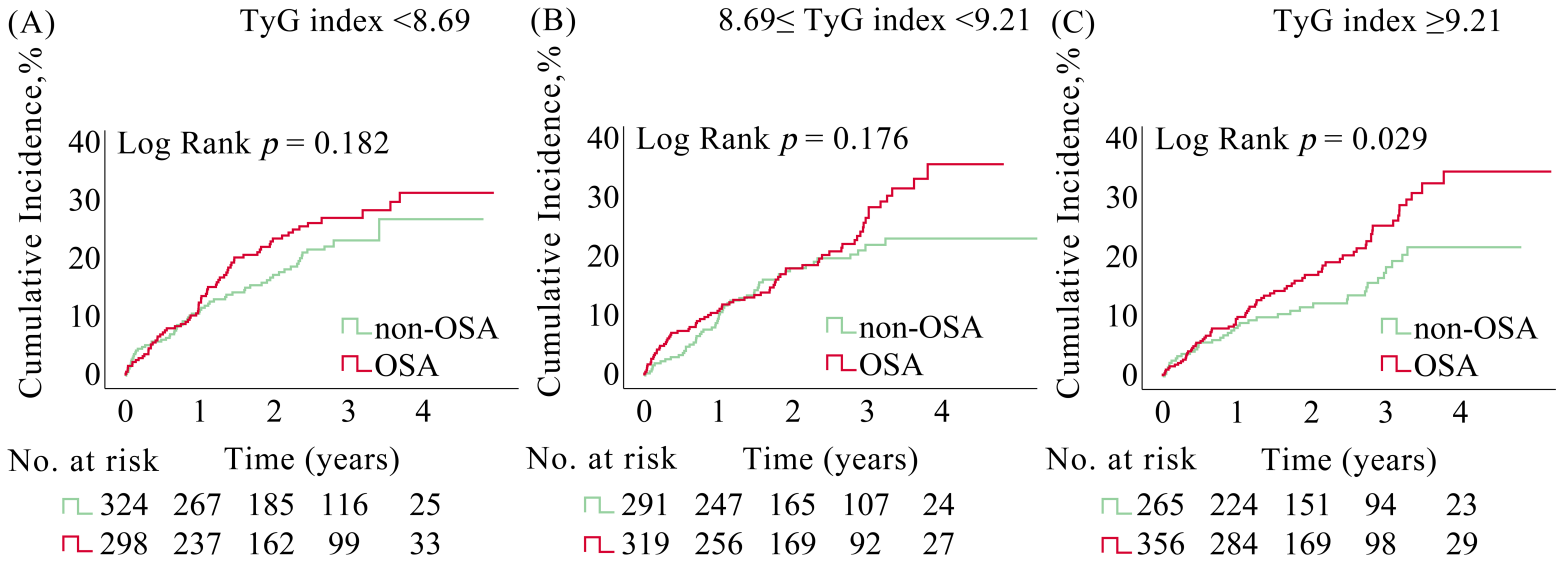
**Kaplan-Meier curves for MACCE in ACS patients with and without OSA stratified by TyG index**. Kaplan-Meier estimates for MACCE in ACS patients from the (A) low TyG group, (B) moderate TyG group, and (C) high TyG group. ACS, acute coronary syndrome; MACCE, major adverse cardiovascular and cerebrovascular events; OSA, obstructive sleep apnea; TyG, triglyceride glucose.

After a median follow-up of 35.1 (19.0–43.5) months, the association between OSA and cardiovascular event risk was further assessed utilizing Cox regression analysis. In the unadjusted model, OSA in the high TyG group (≥9.21) was markedly linked to an elevated risk of MACCE (hazard ratio [HR]: 1.530; 95% confidence interval [CI]: 1.041–2.249; *p* = 0.030) and hospitalization for UA (HR = 1.770; 95% CI: 1.091–2.872; *p* = 0.021). In the fully adjusted model, OSA in the high TyG group (≥9.21) remained significantly associated with elevated MACCE risks (adjusted HR [aHR]: 1.556; 95% CI: 1.040–2.326; *p *= 0.031) and hospitalization for UA (aHR: 1.785; 95% CI: 1.072–2.971; *p *= 0.026). However, no statistically significant association between OSA and MACCE was observed in the moderate or low TyG groups (Fig. 2 and Table 3). The crude event counts for all outcomes are presented in **Supplementary Tables 3,4**.

**Table 3.** **Cox regression analysis of the association between OSA and cardiovascular event risk across TyG index categories**.

		Unadjusted		Partially adjusted*		Fully adjusted†	
		HR (95% CI)	*p* value	HR (95% CI)	*p* value	HR (95% CI)	*p* value
MACCE							
	TyG index <8.69	1.259 (0.897–1.768)	0.183	1.233 (0.877–1.735)	0.228	1.068 (0.737–1.546)	0.729
	8.69 ≤ TyG index < 9.21	1.276 (0.896–1.818)	0.177	1.271 (0.889–1.819)	0.189	1.186 (0.810–1.735)	0.381
	TyG index ≥9.21	1.530 (1.041–2.249)	0.030	1.549 (1.053–2.280)	0.026	1.556 (1.040–2.326)	0.031
Cardiovascular death							
	TyG index <8.69	3.341 (0.904–12.342)	0.070	3.238 (0.872–12.021)	0.079	3.851 (0.950–15.608)	0.059
	8.69 ≤ TyG index < 9.21	0.619 (0.175–2.193)	0.457	0.532 (0.150–1.887)	0.328	0.152 (0.027–1.105)	0.062
	TyG index ≥9.21	0.768 (0.222–2.654)	0.677	0.681 (0.195–2.375)	0.547	0.828 (0.205–3.339)	0.791
Myocardial infarction							
	TyG index <8.69	0.821 (0.285–2.365)	0.714	0.802 (0.277–2.319)	0.684	0.681 (0.206–2.257)	0.530
	8.69 ≤ TyG index < 9.21	2.130 (0.740–6.130)	0.161	2.121 (0.734–6.129)	0.165	1.660 (0.538–5.121)	0.378
	TyG index ≥9.21	1.093 (0.415–2.880)	0.858	1.069 (0.404–2.831)	0.893	0.913 (0.318–2.620)	0.865
Stroke							
	TyG index <8.69	0.662 (0.241–1.821)	0.424	0.679 (0.245–1.878)	0.456	0.530 (0.166–1.688)	0.282
	8.69 ≤ TyG index < 9.21	1.900 (0.572–6.311)	0.295	1.842 (0.552–6.145)	0.320	1.687 (0.466–6.109)	0.426
	TyG index ≥9.21	1.560 (0.533–4.568)	0.417	1.551 (0.527–4.562)	0.426	2.040 (0.630–6.610)	0.234
Hospitalization for UA							
	TyG index <8.69	1.274 (0.853–1.902)	0.237	1.242 (0.830–1.859)	0.292	1.023 (0.660–1.584)	0.920
	8.69 ≤ TyG index < 9.21	1.177 (0.775–1.787)	0.446	1.225 (0.802–1.870)	0.348	1.186 (0.761–1.847)	0.451
	TyG index ≥9.21	1.770 (1.091–2.872)	0.021	1.822 (1.120–2.963)	0.016	1.785 (1.072–2.971)	0.026
Hospitalization for HF							
	TyG index <8.69	0.966 (0.193–4.825)	0.966	0.836 (0.166–4.221)	0.828	1.164 (0.122–11.080)	0.895
	8.69 ≤ TyG index < 9.21	1.274 (0.285–5.697)	0.751	1.281 (0.286–5.740)	0.746	0.805 (0.071–9.084)	0.861
	TyG index ≥9.21	1.058 (0.237–4.728)	0.942	0.721 (0.158–3.296)	0.673	1.127 (0.197–6.455)	0.894
Ischemia-driven revascularization							
	TyG index <8.69	1.277 (0.748–2.181)	0.37	1.231 (0.720–2.106)	0.448	0.940 (0.523–1.693)	0.838
	8.69 ≤ TyG index < 9.21	1.277 (0.752–2.168)	0.365	1.349 (0.789–2.307)	0.273	1.286 (0.727–2.276)	0.387
	TyG index ≥9.21	1.976 (1.009–3.856)	0.047	2.014 (1.028–3.947)	0.041	2.013 (0.973–4.164)	0.059
Composite for cardiovascular death, myocardial infarction, or ischemic stroke							
	TyG index <8.69	1.099 (0.591–2.042)	0.766	1.100 (0.590–2.051)	0.764	0.970 (0.484–1.944)	0.932
	8.69 ≤ TyG index < 9.21	1.569 (0.807–3.050)	0.184	1.495 (0.767–2.913)	0.237	1.073 (0.521–2.212)	0.849
	TyG index ≥9.21	1.301 (0.685–2.470)	0.421	1.259 (0.661–2.397)	0.484	1.318 (0.668–2.602)	0.426
Composite for cardiac events§							
	TyG index <8.69	1.348 (0.942–1.927)	0.102	1.309 (0.913–1.876)	0.142	1.113 (0.753–1.645)	0.590
	8.69 ≤ TyG index < 9.21	1.203 (0.835–1.732)	0.322	1.212 (0.837–1.753)	0.309	1.133 (0.766–1.676)	0.532
	TyG index ≥9.21	1.513 (1.002–2.285)	0.049	1.534 (1.014–2.321)	0.043	1.490 (0.968–2.295)	0.070
All repeat revascularization							
	TyG index <8.69	1.130 (0.705–1.811)	0.613	1.089 (0.679–1.749)	0.723	0.883 (0.525–1.483)	0.637
	8.69 ≤ TyG index < 9.21	1.332 (0.852–2.083)	0.209	1.359 (0.865–2.134)	0.184	1.270 (0.786–2.051)	0.328
	TyG index ≥9.21	1.279 (0.786–2.083)	0.322	1.298 (0.795–2.119)	0.297	1.166 (0.696–1.953)	0.560

*Adjusted for age and sex. †Adjusted for age, sex, body mass index (BMI), estimated glomerular filtration rate (eGFR), left ventricular ejection fraction, diabetes, hypertension, prior stroke, prior myocardial infarction, smoking, diagnosis, presence of heart failure (HF), coronary artery bypass grafting (CABG), P2Y12 inhibitors, and β-blockers. §Include cardiovascular death, myocardial infarction, ischemia-driven revascularization, or hospitalization for UA or HF. CI, confidence interval; HF, heart failure; HR, hazard ratio; MACCE, major adverse cardiovascular and cerebrovascular events; OSA, obstructive sleep apnea; TyG, triglyceride glucose; UA, unstable angina.

## 4. Discussion

This study reveals that in ACS patients, the co-presence of OSA and a high TyG index significantly elevates the risks of MACCE and hospitalization for UA. Moreover, it is the first systematic assessment of the OSA’s impact on cardiovascular outcomes in ACS patients categorized by TyG index levels.

TyG, as a non-invasive surrogate marker for insulin resistance, reflects the degree of insulin resistance [26]. Insulin resistance is defined as a reduced responsiveness of target tissues to elevated insulin levels, characterized by impaired glucose uptake, decreased glycogen synthesis, and diminished lipid oxidation capacity [27]. This condition consequently heightens the risk of oxidative stress and inflammation.β-blockers represent a cornerstone therapy in ACS management [28]. Despite their significant cardioprotective effects in reducing myocardial oxygen consumption and preventing arrhythmias, certain β-blockers may compromise metabolic function through the inhibition of lipolysis and glucose metabolism, thereby potentially worsening insulin resistance [29, 30]. Since the TyG index is a reliable surrogate marker for insulin resistance, the use of β-blockers in ACS patients with elevated TyG index should be approached with caution. The adverse metabolic effects of β-blockers may interact with insulin resistance caused by OSA, potentially increasing cardiovascular risk in this population.

OSA induces oxidative stress through intermittent hypoxia, which subsequently triggers inflammation and endothelial injury—key factors in the progression of atherosclerosis and subsequent cardiovascular disease [31, 32]. Intermittent hypoxia also activates the pro-inflammatory transcription factor nuclear factor-κB and upregulates downstream inflammatory cytokines such as interleukin-8, facilitating leukocyte migration and the adhesion molecules expression, thereby intensifying vascular inflammation [33, 34]. Moreover, intermittent hypoxia, hypercapnia, and sleep fragmentation disrupt the gut microbiota, compromise intestinal epithelial integrity, and enhance local and systemic inflammatory responses, which ultimately increase the risk of cardiovascular disease and metabolic abnormalities, such as insulin resistance [35, 36]. Our study reveals that in patients with high TyG index, the AHI, ODI, and time spent with oxygen saturation below 90% are significantly prolonged, while low-grade inflammation, marked by elevated hs-CRP levels, is significantly higher, suggesting a notable increase in OSA severity with elevated TyG index levels.

Nevertheless, existing literature lacks investigations into the association between OSA and cardiovascular outcomes in ACS patients categorized by TyG index. In a study of 154 patients with type 2 diabetes, Ding and Jiang [37] reported that in those aged ≥50 years, TyG mediated the effect of OSA-induced arterial stiffness, accounting for 33.42% of the total effect. Although limitations in sample size and the cross-sectional nature of this study constrain the robustness and generalizability of its conclusions-particularly given its focus on arterial stiffness rather than prognostic outcomes-these findings suggest a potential synergistic relationship between OSA and insulin resistance in elevating cardiovascular risk in ACS patients. Indeed, our findings confirm this hypothesis. In our study, OSA significantly increased the risk of MACCE and hospitalization for UA exclusively in the high TyG subgroup of ACS patients. Thus, we posit that OSA exerts a synergistic effect in ACS patients with elevated TyG index and recommend routine screening for OSA in this high-risk population, particularly among those with higher TyG indices.

Our findings hold significant clinical relevance and have the potential to advance risk stratification and management strategies for ACS patients. Screening for OSA has not been a routine component of the risk assessment protocol for ACS patients. However, our analysis suggests that the integration of OSA screening, particularly in ACS patients with an elevated TyG index, can effectively identify individuals at higher cardiovascular risk and enable personalized therapeutic interventions targeting both metabolic dysfunction and OSA, thereby mitigating composite cardiovascular risk. Furthermore, β-blocker therapy for ACS patients may necessitate individualized modification based on TyG index levels and OSA to achieve a more precise therapeutic approach. Future research should further investigate the efficacy of interventions targeting OSA and metabolic dysfunction in high-risk ACS patients, thereby establishing a robust foundation for more targeted ACS management strategies.

The strengths of this study include a large, well-characterized cohort from the OSA-ACS study, enabling stratified analysis based on TyG index. Moreover, the utilization of comprehensive clinical data and standardized diagnosis criteria for OSA enhances the reliability of our findings. These strengths enable us to investigate the interplay between metabolic and sleep-related factors, providing nuanced insights into their synergistic effects on cardiovascular outcomes and supporting the clinical validity and applicability of our conclusions.

### Limitations

This study has several limitations. First, as a single-center cohort study, our conclusions lack validation from external datasets, potentially affecting the robustness and generalizability of our findings to broader populations. Second, although the TyG index has been validated in multiple studies as a reliable surrogate marker for insulin resistance, this study did not employ the gold-standard method for measuring insulin resistance, which may somewhat weaken the support for our conclusions. Third, OSA was assessed using portable polysomnography, which may have reduced accuracy compared to standard laboratory-based polysomnography, potentially affecting the precision of certain sleep-related parameters. Fourth, insufficient documentation of adherence to lifestyle modifications during the follow-up period prevented adequate adjustment for these potential confounders. Furthermore, low adherence to CPAP therapy following discharge significantly constrained our ability to evaluate treatment-specific effects.

## 5. Conclusions

This post-hoc analysis reveals that in ACS patients with high TyG index, OSA significantly increases cardiovascular event risk, suggesting a potential synergistic effect between metabolic dysregulation and OSA that intensifies cardiovascular burden in ACS patients. These findings underscore the value of OSA screening in this high-risk population to optimize risk stratification and guide therapeutic decision-making, ultimately improving long-term cardiovascular outcomes and advancing individualized management strategies for ACS patients.

## Availability of Data and Materials

The data regarding this article will be shared by the corresponding author upon reasonable request.

## Supplementary Material

Supplementary material associated with this article can be found, in the online version, at https://doi.org/10.31083/RCM36205.
