# Supplementary material for: Impact of Obstructive Sleep Apnea and Triglyceride Glucose Index on Cardiovascular Events in Acute Coronary Syndrome Patients: A Post-Hoc Analysis of the OSA–ACS Study
Source: Rev Cardiovasc Med. 2025 May 21;26(5):36205. doi: 10.31083/RCM36205 (PMC12135658; doi:10.31083/RCM36205)
Supplement: Supplementary file 1 [file 2153-8174-26-5-36205-s1.docx]

**Supplemental Table S1. Baseline Clinical Characteristics of OSA versus non-OSA group overall and in patients of TyG index ≥9.21**

|  | **All (N=1853)** | | | **TyG index ≥9.21 (N = 621)** | | |
| --- | --- | --- | --- | --- | --- | --- |
|  | **OSA (n=973)** | **Non-OSA (n=880)** | ***p* value** | **OSA (n=356)** | **Non-OSA(n=265)** | ***p* value** |
| **Demographics** |  |  |  |  |  |  |
| Age, years | 56.5 ± 10.6 | 56.2 ± 10.4 | 0.500 | 53.9 ± 10.7 | 53.5 ± 10.4 | 0.629 |
| Male | 853(87.7） | 718(81.6) | <0.001 | 314(88.2) | 221(83.4) | 0.086 |
| BMI, kg/m^2^ | 28.0 ± 3.5 | 26.0 ± 3.4 | <0.001 | 28.6 ± 3.4 | 27.0 ± 3.2 | <0.001 |
| Waist-to-hip ratio | 0.99(0.96-1.02) | 0.97(0.94-1.00) | <0.001 | 0.99(0.97-1.03) | 0.98(0.95-1.02) | <0.001 |
| Neck circumference, cm | 42(39-44) | 40(38-42) | <0.001 | 42(40-45) | 41(39-43) | <0.001 |
| Systolic BP, mmHg | 127(117-139) | 126(117-138) | 0.398 | 127(118-138) | 127(117-140) | 0.804 |
| Diastolic BP, mmHg | 77(70-86) | 75(69-83) | <0.001 | 79(70-88) | 78(70-86) | 0.208 |
| **Medical History** |  |  |  |  |  |  |
| Diabetes | 307(31.6) | 283(32.2) | 0.779 | 173(48.6) | 127(47.9) | 0.869 |
| Hypertension | 663(68.1) | 535(60.8) | <0.001 | 256(71.9) | 175(66.0) | 0.116 |
| Hyperlipidemia | 325(33.4) | 282(32.0) | 0.534 | 139(39.0) | 97(36.6) | 0.535 |
| Family history of premature CAD | 51(5.2) | 50(5.7) | 0.677 | 18(5.1) | 18(6.8) | 0.360 |
| Prior stroke | 116(11.9) | 82(9.3) | 0.070 | 35(9.8) | 22(8.3) | 0.514 |
| Prior myocardial infarction | 171(17.6) | 131(14.9) | 0.118 | 61(17.1) | 36(13.6) | 0.228 |
| Prior PCI | 225(23.1) | 158(18.0) | 0.006 | 85(23.9) | 49(18.5) | 0.107 |
| Smoking |  |  | 0.416 |  |  | 0.743 |
| No | 321(33.0) | 311(35.3) |  | 113(31.7) | 83(31.3) |  |
| Current | 475(48.8) | 403(45.8) |  | 188(52.8) | 135(50.9) |  |
| Previous | 177(18.2) | 166(18.9) |  | 55(15.4) | 47(17.7) |  |
| Drinking |  |  | 0.005 |  |  | 0.019 |
| No | 565(58.1) | 572(65.0) |  | 194(54.5) | 172(64.9) |  |
| Current | 345(35.5) | 270(30.7) |  | 143(40.2) | 86(32.5) |  |
| Previous | 63(6.5) | 38(4.3) |  | 19(5.3) | 7(2.6) |  |
| Presence of HF | 9(0.9) | 2(0.2) | 0.051 | 4(1.1) | 2(0.8) | 0.960 |
| **Baseline Tests** |  |  |  |  |  |  |
| eGFR, mL/min/1.73 m^2^ | 103.1(87.5-119.0) | 107.3(91.0-123.7) | 0.002 | 102.7 (87.7-119.2) | 107.1(89.3-121.0) | 0.350 |
| Hs-CRP, mg/L | 2.5(1.0-7.3) | 1.5(0.6-4.3) | <0.001 | 3.5(1.3-8.4) | 2.3(0.9-5.5)) | <0.001 |
| LVEF, % | 61(55-65) | 62(58-66) | 0.006 | 60(55-65) | 62(57-66) | 0.115 |

BMI, body mass index; BP, blood pressure; CAD, coronary artery disease; eGFR, glomerular filtration rate; HF, heart failure; Hs-CRP, high-sensitivity C-reactive protein; IQR, interquartile range; LVEF, left ventricular ejection fraction; OSA, obstructive sleep apnea; PCI, percutaneous coronary intervention; SD, standard deviation; TyG, triglyceride glucose.

**Supplemental Table S2. Clinical Presentations and Management of OSA versus non-OSA group overall and** **in patients of TyG index ≥9.21**

|  | **All (N=1853)** | | | **TyG index ≥9.21 (N = 621)** | | |
| --- | --- | --- | --- | --- | --- | --- |
|  | **OSA (n=973)** | **Non-OSA (n=880)** | ***p* value** | **OSA (n=356)** | **Non-OSA(n=265)** | ***p* value** |
| **Diagnosis** |  |  | 0.038 |  |  | 0.198 |
| STEMI | 242(24.9) | 176(20.0) |  | 86(24.2) | 50(18.9) |  |
| NSTEMI | 183(18.8) | 168(19.1) |  | 76(21.3) | 53(20.0) |  |
| UA | 548(56.3) | 536(60.9) |  | 194(54.5) | 162(61.1) |  |
| **Procedures** |  |  |  |  |  |  |
| Coronary angiography | 952(97.8) | 854(97.0) | 0.276 | 350(98.3) | 259(97.7) | 0.604 |
| PCI | 641(65.9) | 523(59.4) | 0.004 | 245(68.8) | 169(63.8) | 0.187 |
| DES use | 556(86.7) | 451(86.2) | 0.801 | 210(85.7) | 146(86.4) | 0.846 |
| Baseline TIMI 0 or 1 | 233(36.3) | 171(32.7) | 0.193 | 94(38.4) | 62(36.7) | 0.729 |
| CABG | 57(5.9) | 70(8.0) | 0.074 | 25(7.0) | 21(7.9) | 0.671 |
| **Sleep Study** |  |  |  |  |  |  |
| AHI, events·h^−1^ | 29.0(20.7-42.0) | 7.7(4.2-10.8) | <0.001 | 31.9(22.2-47.2) | 7.6(4.3-11.1) | <0.001 |
| ODI, events·h^−1^ | 27.5(20.2-39.6) | 8.6(4.9-11.9) | <0.001 | 29.7(21.1-44.5) | 8.8(5.3-12.0) | <0.001 |
| Nadir SaO_2_, % | 83(77-86) | 87(84-90) | <0.001 | 82(76-86) | 87(84-89) | <0.001 |
| Mean SaO_2_, % | 93(92-94) | 94(93-95) | <0.001 | 93(92-94) | 94(93-95) | <0.001 |
| Time with SaO_2_<90%, % | 6.0(2.0-15.0) | 0.6(0.1-3.0) | <0.001 | 6.0(2.0-17.0) | 1.0(0.1-3.0) | <0.001 |
| Epworth Sleepiness Scale | 8.0(5.0-12.0) | 6.0(3.0-10.0) | <0.001 | 9.0(6.0-13.0) | 8.0(4.0-11.0) | 0.005 |
| **Medications on Discharge** |  |  |  |  |  |  |
| Aspirin | 947(97.3) | 858(97.5) | 0.816 | 346(97.2) | 262(98.9) | 0.149 |
| P2Y_12_ inhibitors | 901(92.6) | 801(91.0) | 0.215 | 335(94.1) | 242(91.3) | 0.182 |
| β-Blockers | 765(78.6) | 664(75.5) | 0.105 | 291(81.7) | 208(78.5) | 0.313 |
| ACEIs/ARBs | 641(65.9) | 510(58.0) | <0.001 | 231(64.9) | 160(60.4) | 0.250 |
| Statins | 958(98.5) | 867(98.5) | 0.910 | 347(97.5) | 261(98.5) | 0.380 |

ACEI, angiotensin-converting enzymes inhibitor; AHI, apnea-hypopnea index; ARB, angiotensin receptor blocker; CABG, coronary artery bypass grafting; DES, drug eluting stent; IQR, interquartile range; NSTEMI, non-ST-segment elevation myocardial infarction; ODI, oxygen desaturation index; OSA, obstructive sleep apnea; PCI, percutaneous coronary intervention; SaO_2_, arterial oxygen saturation; SD, standard deviation; STEMI, ST-segment-elevation myocardial infarction; TIMI, thrombolysis in myocardial infarction; TyG, triglyceride glucose; UA, unstable angina.

**Supplemental Table S3. Crude number of all events overall and** **by TyG index categories**

|  | **All**  **(N=1853)** | **TyG index <8.69**  **(N = 622)** | **8.69 ≤TyG index<9.21**  **(N =610)** | **TyG index ≥9.21**  **(N = 621)** | ***p***  **Value** |
| --- | --- | --- | --- | --- | --- |
| MACCE | 373(20.1) | 134(21.5) | 125(20.5) | 114(18.4) | 0.361 |
| Cardiovascular death | 32(1.7) | 12(1.9) | 10(1.6) | 10(1.6) | 0.893 |
| Myocardial infarction | 47(2.5) | 14(2.3) | 16(2.6) | 17(2.7) | 0.850 |
| Stroke | 43(2.3) | 16(2.6) | 12(2.0) | 15(2.4) | 0.765 |
| Hospitalization for UA | 261(14.1) | 96(15.4) | 89(14.6) | 76(12.2) | 0.245 |
| Hospitalization for HF | 20(1.1) | 6(1.0) | 7(1.1) | 7(1.1) | 0.943 |
| Ischemia-driven revascularization | 152(8.2) | 54(8.7) | 56(9.2) | 42(6.8) | 0.263 |
| Composite of major cardiovascular events | 117(6.3) | 40(6.4) | 37(6.1) | 40(6.4) | 0.954 |
| Composite for cardiac events | 337(18.2) | 121(19.5) | 117(19.2) | 99(15.9) | 0.204 |
| All repeat revascularization | 217(11.7) | 69(11.1) | 79(13.0) | 69(11.1) | 0.508 |

Composite end point of major cardiovascular events included cardiovascular death, myocardial infarction, and stroke; Composite for cardiac events included cardiovascular death, myocardial infarction, ischemia-driven revascularization, or hospitalization for UA or HF. HF, heart failure; MACCE, major adverse cardiovascular and cerebrovascular event; TyG, triglyceride glucose; UA, unstable angina.

**Supplemental Table S4. Crude Number of all Events of OSA versus non-OSA group overall and in patients of TyG index ≥9.21**

|  | **All (N=1853)** | | | **TyG index ≥9.21 (N = 621)** | | |
| --- | --- | --- | --- | --- | --- | --- |
|  | **OSA (n=973)** | **Non-OSA (n=880)** | ***p* value** | **OSA (n=356)** | **Non-OSA(n=265)** | ***p* value** |
| MACCE | 216(22.2) | 157(17.8) | 0.019 | 74(20.8) | 40(15.1) | 0.070 |
| Cardiovascular death | 18(1.8) | 14(1.6) | 0.669 | 5(1.4) | 5(1.9) | 0.637 |
| Myocardial infarction | 27(2.8) | 20(2.3) | 0.492 | 10(2.8) | 7(2.6) | 0.899 |
| Stroke | 24(2.5) | 19(2.2) | 0.661 | 10(2.8) | 5(1.9) | 0.459 |
| Hospitalization for UA | 152(15.6) | 109(12.4) | 0.046 | 52(14.6) | 24(9.1) | 0.037 |
| Hospitalization for HF | 11(1.1) | 9(1.0) | 0.823 | 4(1.1) | 3(1.1) | >0.999 |
| Ischemia-driven revascularization | 91(9.4) | 61(6.9) | 0.058 | 30(8.4) | 12(4.5) | 0.056 |
| Composite of major cardiovascular events | 68(7.0) | 49(5.6) | 0.209 | 25(7.0) | 15(5.7) | 0.494 |
| Composite for cardiac events | 195(20.0) | 142(16.1) | 0.030 | 64(18.0) | 35(13.2) | 0.108 |
| All repeat revascularization | 124(12.7) | 93(10.6) | 0.146 | 43(12.1) | 26(9.8) | 0.374 |

Composite end point of major cardiovascular events included cardiovascular death, myocardial infarction, and stroke; Composite for cardiac events included cardiovascular death, myocardial infarction, ischemia-driven revascularization, or hospitalization for UA or HF. HF, heart failure; MACCE, major adverse cardiovascular and cerebrovascular event; OSA, obstructive sleep apnea; TyG, triglyceride glucose; UA, unstable angina.
